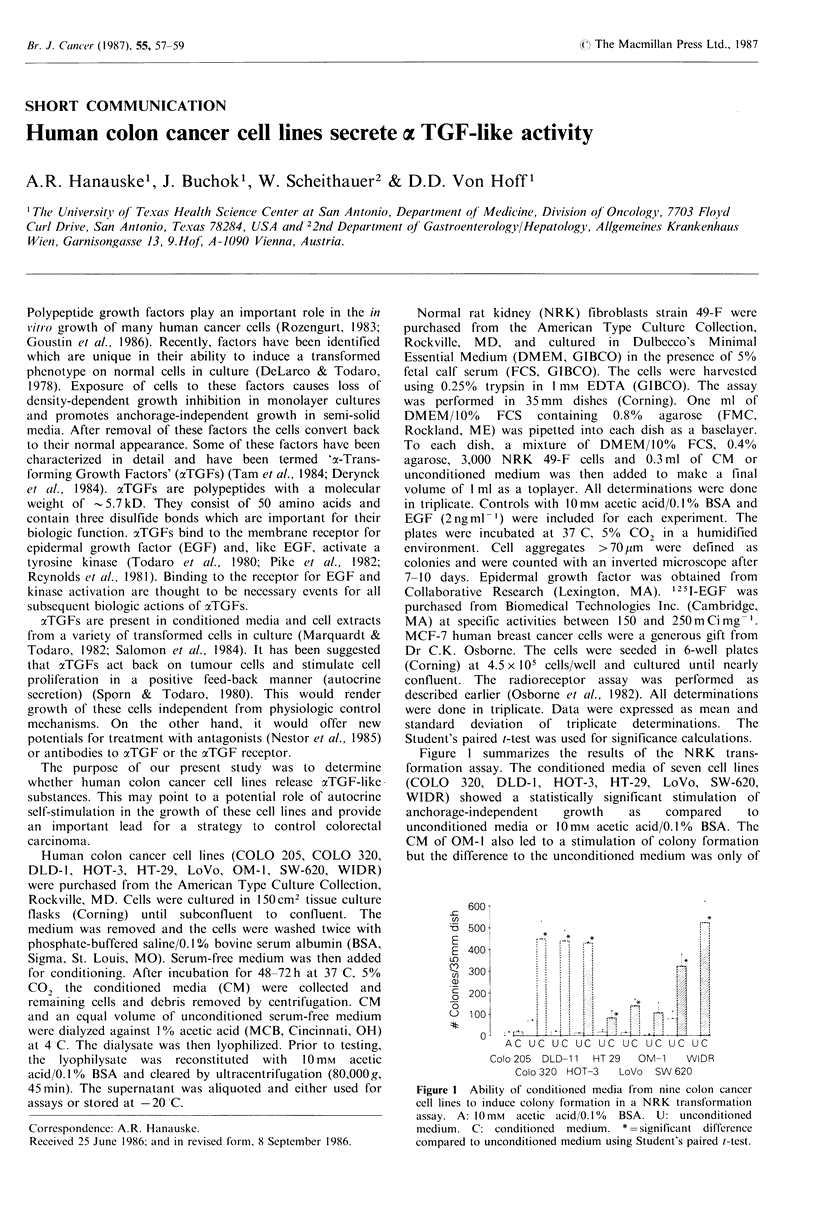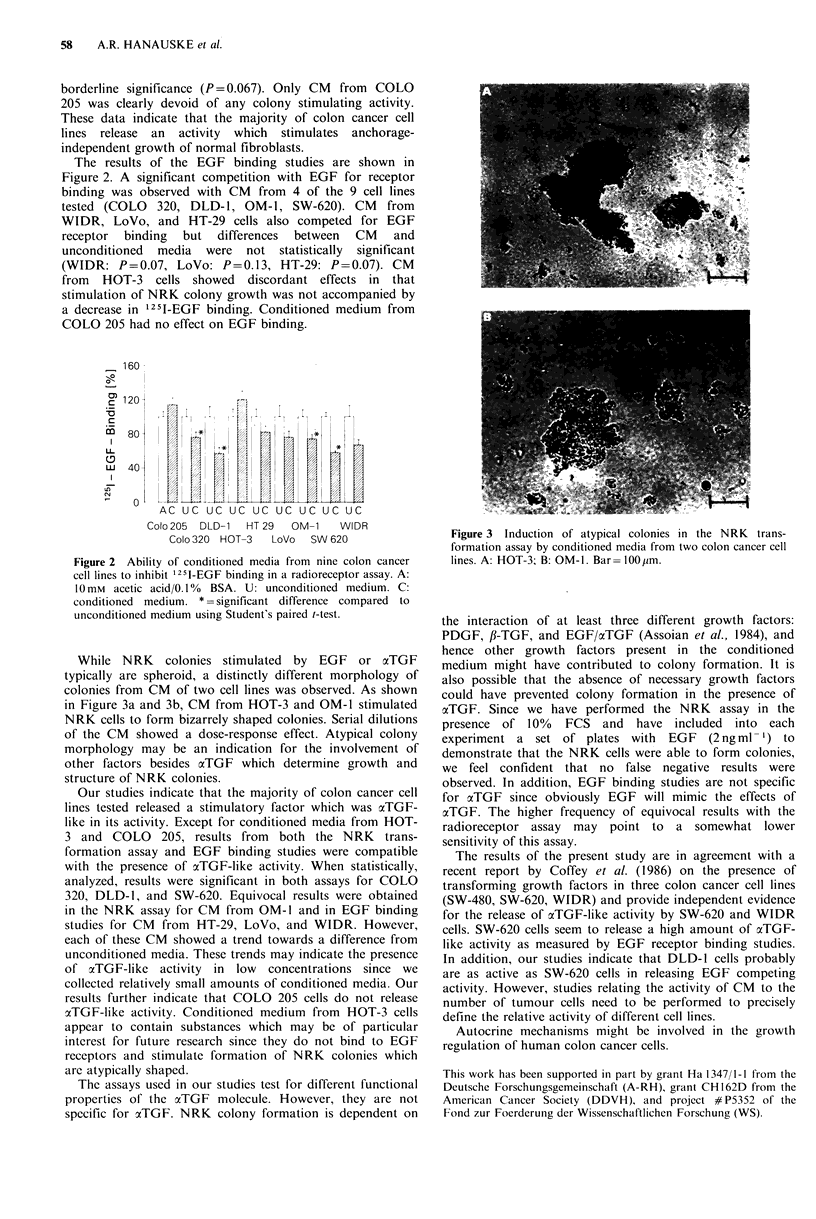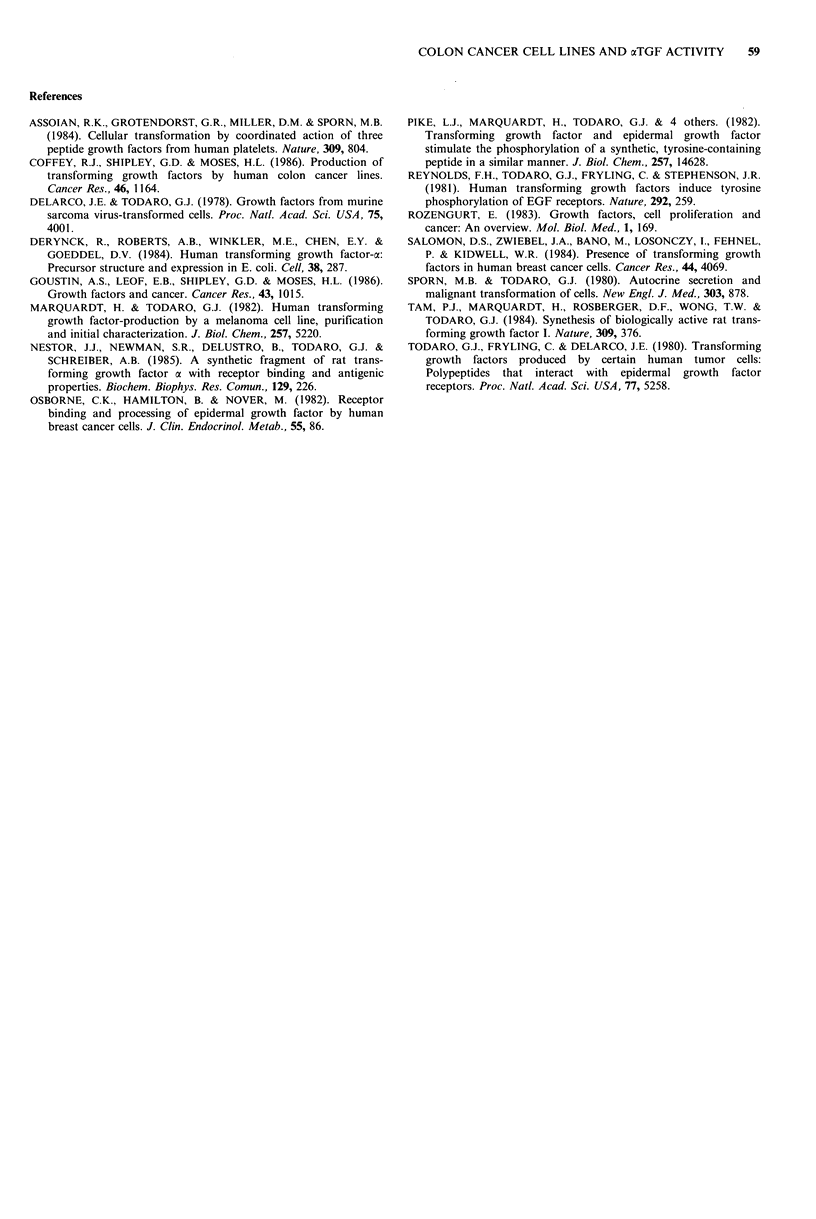# Human colon cancer cell lines secrete alpha TGF-like activity.

**DOI:** 10.1038/bjc.1987.12

**Published:** 1987-01

**Authors:** A. R. Hanauske, J. Buchok, W. Scheithauer, D. D. Von Hoff

## Abstract

**Images:**


*Br. J. Cancer* (1987), **55**, 57–59 © The Macmillan Press Ltd., 1987

SHORT COMMUNICATION

### Human colon cancer cell lines secrete α TGF-like activity

A.R. Hanauske[1], J. Buchok[1], W. Scheithauer[2] & D.D. Von Hoff[1]

[1]*The University of Texas Health Science Center at San Antonio, Department of Medicine, Division of Oncology, 7703 Floyd Curl Drive, San Antonio, Texas 78284, USA and* [2]*2nd Department of Gastroenterology/Hepatology, Allgemeines Krankenhaus Wien, Garnisongasse 13, 9.Hof, A-1090 Vienna, Austria.*

Polypeptide growth factors play an important role in the *in vitro* growth of many human cancer cells (Rozengurt, 1983; Goustin *et al.*, 1986). Recently, factors have been identified which are unique in their ability to induce a transformed phenotype on normal cells in culture (DeLarco & Todaro, 1978). Exposure of cells to these factors causes loss of density-dependent growth inhibition in monolayer cultures and promotes anchorage-independent growth in semi-solid media. After removal of these factors the cells convert back to their normal appearance. Some of these factors have been characterized in detail and have been termed 'α-Transforming Growth Factors' (αTGFs) (Tam *et al.*, 1984; Derynck *et al.*, 1984). αTGFs are polypeptides with a molecular weight of ~5.7kD. They consist of 50 amino acids and contain three disulfide bonds which are important for their biologic function. αTGFs bind to the membrane receptor for epidermal growth factor (EGF) and, like EGF, activate a tyrosine kinase (Todaro *et al.*, 1980; Pike *et al.*, 1982; Reynolds *et al.*, 1981). Binding to the receptor for EGF and kinase activation are thought to be necessary events for all subsequent biologic actions of αTGFs.

αTGFs are present in conditioned media and cell extracts from a variety of transformed cells in culture (Marquardt & Todaro, 1982; Salomon *et al.*, 1984). It has been suggested that αTGFs act back on tumour cells and stimulate cell proliferation in a positive feed-back manner (autocrine secretion) (Sporn & Todaro, 1980). This would render growth of these cells independent from physiologic control mechanisms. On the other hand, it would offer new potentials for treatment with antagonists (Nestor *et al.*, 1985) or antibodies to αTGF or the αTGF receptor.

The purpose of our present study was to determine whether human colon cancer cell lines release αTGF-like substances. This may point to a potential role of autocrine self-stimulation in the growth of these cell lines and provide an important lead for a strategy to control colorectal carcinoma.

Human colon cancer cell lines (COLO 205, COLO 320, DLD-1, HOT-3, HT-29, LoVo, OM-1, SW-620, WIDR) were purchased from the American Type Culture Collection, Rockville, MD. Cells were cultured in $150\,cm^2$ tissue culture flasks (Corning) until subconfluent to confluent. The medium was removed and the cells were washed twice with phosphate-buffered saline/0.1% bovine serum albumin (BSA, Sigma, St. Louis, MO). Serum-free medium was then added for conditioning. After incubation for 48–72h at 37°C, 5% $CO_2$ the conditioned media (CM) were collected and remaining cells and debris removed by centrifugation. CM and an equal volume of unconditioned serum-free medium were dialyzed against 1% acetic acid (MCB, Cincinnati, OH) at 4°C. The dialysate was then lyophilized. Prior to testing, the lyophilysate was reconstituted with 10mM acetic acid/0.1% BSA and cleared by ultracentrifugation (80,000*g*, 45min). The supernatant was aliquoted and either used for assays or stored at −20°C.

Normal rat kidney (NRK) fibroblasts strain 49-F were purchased from the American Type Culture Collection, Rockville, MD, and cultured in Dulbecco's Minimal Essential Medium (DMEM, GIBCO) in the presence of 5% fetal calf serum (FCS, GIBCO). The cells were harvested using 0.25% trypsin in 1mM EDTA (GIBCO). The assay was performed in 35mm dishes (Corning). One ml of DMEM/10% FCS containing 0.8% agarose (FMC, Rockland, ME) was pipetted into each dish as a baselayer. To each dish, a mixture of DMEM/10% FCS, 0.4% agarose, 3,000 NRK 49-F cells and 0.3ml of CM or unconditioned medium was then added to make a final volume of 1ml as a toplayer. All determinations were done in triplicate. Controls with 10mM acetic acid/0.1% BSA and EGF ($2\,ng\,ml^{-1}$) were included for each experiment. The plates were incubated at 37°C, 5% $CO_2$ in a humidified environment. Cell aggregates $>70\,\mu m$ were defined as colonies and were counted with an inverted microscope after 7–10 days. Epidermal growth factor was obtained from Collaborative Research (Lexington, MA). $^{125}$I-EGF was purchased from Biomedical Technologies Inc. (Cambridge, MA) at specific activities between 150 and $250\,mCi\,mg^{-1}$. MCF-7 human breast cancer cells were a generous gift from Dr C.K. Osborne. The cells were seeded in 6-well plates (Corning) at $4.5\times10^5$ cells/well and cultured until nearly confluent. The radioreceptor assay was performed as described earlier (Osborne *et al.*, 1982). All determinations were done in triplicate. Data were expressed as mean and standard deviation of triplicate determinations. The Student's paired *t*-test was used for significance calculations.

Figure 1 summarizes the results of the NRK transformation assay. The conditioned media of seven cell lines (COLO 320, DLD-1, HOT-3, HT-29, LoVo, SW-620, WIDR) showed a statistically significant stimulation of anchorage-independent growth as compared to unconditioned media or 10mM acetic acid/0.1% BSA. The CM of OM-1 also led to a stimulation of colony formation but the difference to the unconditioned medium was only of

**Figure 1** Ability of conditioned media from nine colon cancer cell lines to induce colony formation in a NRK transformation assay. A: 10mM acetic acid/0.1% BSA. U: unconditioned medium. C: conditioned medium. * = significant difference compared to unconditioned medium using Student's paired *t*-test.

Correspondence: A.R. Hanauske.

Received 25 June 1986; and in revised form, 8 September 1986.

borderline significance ($P=0.067$). Only CM from COLO 205 was clearly devoid of any colony stimulating activity. These data indicate that the majority of colon cancer cell lines release an activity which stimulates anchorage-independent growth of normal fibroblasts.

The results of the EGF binding studies are shown in Figure 2. A significant competition with EGF for receptor binding was observed with CM from 4 of the 9 cell lines tested (COLO 320, DLD-1, OM-1, SW-620). CM from WIDR, LoVo, and HT-29 cells also competed for EGF receptor binding but differences between CM and unconditioned media were not statistically significant (WIDR: $P=0.07$, LoVo: $P=0.13$, HT-29: $P=0.07$). CM from HOT-3 cells showed discordant effects in that stimulation of NRK colony growth was not accompanied by a decrease in $^{125}$I-EGF binding. Conditioned medium from COLO 205 had no effect on EGF binding.

**Figure 2** Ability of conditioned media from nine colon cancer cell lines to inhibit $^{125}$I-EGF binding in a radioreceptor assay. A: 10 mM acetic acid/0.1% BSA. U: unconditioned medium. C: conditioned medium. * = significant difference compared to unconditioned medium using Student's paired *t*-test.

**Figure 3** Induction of atypical colonies in the NRK transformation assay by conditioned media from two colon cancer cell lines. A: HOT-3; B: OM-1. Bar = 100 μm.

While NRK colonies stimulated by EGF or αTGF typically are spheroid, a distinctly different morphology of colonies from CM of two cell lines was observed. As shown in Figure 3a and 3b, CM from HOT-3 and OM-1 stimulated NRK cells to form bizarrely shaped colonies. Serial dilutions of the CM showed a dose-response effect. Atypical colony morphology may be an indication for the involvement of other factors besides αTGF which determine growth and structure of NRK colonies.

Our studies indicate that the majority of colon cancer cell lines tested released a stimulatory factor which was αTGF-like in its activity. Except for conditioned media from HOT-3 and COLO 205, results from both the NRK transformation assay and EGF binding studies were compatible with the presence of αTGF-like activity. When statistically, analyzed, results were significant in both assays for COLO 320, DLD-1, and SW-620. Equivocal results were obtained in the NRK assay for CM from OM-1 and in EGF binding studies for CM from HT-29, LoVo, and WIDR. However, each of these CM showed a trend towards a difference from unconditioned media. These trends may indicate the presence of αTGF-like activity in low concentrations since we collected relatively small amounts of conditioned media. Our results further indicate that COLO 205 cells do not release αTGF-like activity. Conditioned medium from HOT-3 cells appear to contain substances which may be of particular interest for future research since they do not bind to EGF receptors and stimulate formation of NRK colonies which are atypically shaped.

The assays used in our studies test for different functional properties of the αTGF molecule. However, they are not specific for αTGF. NRK colony formation is dependent on the interaction of at least three different growth factors: PDGF, β-TGF, and EGF/αTGF (Assoian *et al.*, 1984), and hence other growth factors present in the conditioned medium might have contributed to colony formation. It is also possible that the absence of necessary growth factors could have prevented colony formation in the presence of αTGF. Since we have performed the NRK assay in the presence of 10% FCS and have included into each experiment a set of plates with EGF (2 ng ml$^{-1}$) to demonstrate that the NRK cells were able to form colonies, we feel confident that no false negative results were observed. In addition, EGF binding studies are not specific for αTGF since obviously EGF will mimic the effects of αTGF. The higher frequency of equivocal results with the radioreceptor assay may point to a somewhat lower sensitivity of this assay.

The results of the present study are in agreement with a recent report by Coffey *et al.* (1986) on the presence of transforming growth factors in three colon cancer cell lines (SW-480, SW-620, WIDR) and provide independent evidence for the release of αTGF-like activity by SW-620 and WIDR cells. SW-620 cells seem to release a high amount of αTGF-like activity as measured by EGF receptor binding studies. In addition, our studies indicate that DLD-1 cells probably are as active as SW-620 cells in releasing EGF competing activity. However, studies relating the activity of CM to the number of tumour cells need to be performed to precisely define the relative activity of different cell lines.

Autocrine mechanisms might be involved in the growth regulation of human colon cancer cells.

This work has been supported in part by grant Ha 1347/1-1 from the Deutsche Forschungsgemeinschaft (A-RH), grant CH162D from the American Cancer Society (DDVH), and project #P5352 of the Fond zur Foerderung der Wissenschaftlichen Forschung (WS).

### References

ASSOIAN, R.K., GROTENDORST, G.R., MILLER, D.M. & SPORN, M.B. (1984). Cellular transformation by coordinated action of three peptide growth factors from human platelets. *Nature*, **309**, 804.

COFFEY, R.J., SHIPLEY, G.D. & MOSES, H.L. (1986). Production of transforming growth factors by human colon cancer lines. *Cancer Res.*, **46**, 1164.

DELARCO, J.E. & TODARO, G.J. (1978). Growth factors from murine sarcoma virus-transformed cells. *Proc. Natl. Acad. Sci. USA*, **75**, 4001.

DERYNCK, R., ROBERTS, A.B., WINKLER, M.E., CHEN, E.Y. & GOEDDEL, D.V. (1984). Human transforming growth factor-α: Precursor structure and expression in E. coli. *Cell*, **38**, 287.

GOUSTIN, A.S., LEOF, E.B., SHIPLEY, G.D. & MOSES, H.L. (1986). Growth factors and cancer. *Cancer Res.*, **43**, 1015.

MARQUARDT, H. & TODARO, G.J. (1982). Human transforming growth factor-production by a melanoma cell line, purification and initial characterization. *J. Biol. Chem.*, **257**, 5220.

NESTOR, J.J., NEWMAN, S.R., DELUSTRO, B., TODARO, G.J. & SCHREIBER, A.B. (1985). A synthetic fragment of rat transforming growth factor α with receptor binding and antigenic properties. *Biochem. Biophys. Res. Comun.*, **129**, 226.

OSBORNE, C.K., HAMILTON, B. & NOVER, M. (1982). Receptor binding and processing of epidermal growth factor by human breast cancer cells. *J. Clin. Endocrinol. Metab.*, **55**, 86.

PIKE, L.J., MARQUARDT, H., TODARO, G.J. & 4 others. (1982). Transforming growth factor and epidermal growth factor stimulate the phosphorylation of a synthetic, tyrosine-containing peptide in a similar manner. *J. Biol. Chem.*, **257**, 14628.

REYNOLDS, F.H., TODARO, G.J., FRYLING, C. & STEPHENSON, J.R. (1981). Human transforming growth factors induce tyrosine phosphorylation of EGF receptors. *Nature*, **292**, 259.

ROZENGURT, E. (1983). Growth factors, cell proliferation and cancer: An overview. *Mol. Biol. Med.*, **1**, 169.

SALOMON, D.S., ZWIEBEL, J.A., BANO, M., LOSONCZY, I., FEHNEL, P. & KIDWELL, W.R. (1984). Presence of transforming growth factors in human breast cancer cells. *Cancer Res.*, **44**, 4069.

SPORN, M.B. & TODARO, G.J. (1980). Autocrine secretion and malignant transformation of cells. *New Engl. J. Med.*, **303**, 878.

TAM, P.J., MARQUARDT, H., ROSBERGER, D.F., WONG, T.W. & TODARO, G.J. (1984). Synethesis of biologically active rat transforming growth factor I. *Nature*, **309**, 376.

TODARO, G.J., FRYLING, C. & DELARCO, J.E. (1980). Transforming growth factors produced by certain human tumor cells: Polypeptides that interact with epidermal growth factor receptors. *Proc. Natl. Acad. Sci. USA*, **77**, 5258.